# Construction of copy number variation landscape and characterization of associated genes in a Bangladeshi cohort of neurodevelopmental disorders

**DOI:** 10.3389/fgene.2023.955631

**Published:** 2023-03-07

**Authors:** Hosneara Akter, Muhammad Mizanur Rahman, Shaoli Sarker, Mohammed Basiruzzaman, Md. Mazharul Islam, Md. Atikur Rahaman, Md. Ashiquir Rahaman, Tamannyat Binte Eshaque, Nushrat Jahan Dity, Shouvik Sarker, Md. Robed Amin, Mohammad Monir Hossain, Maksuda Lopa, Nargis Jahan, Shafaat Hossain, Amirul Islam, Ashaduzzaman Mondol, Md Omar Faruk, Narayan Saha, Gopen kumar Kundu, Shayla Imam Kanta, Rezaul Karim Kazal, Kanij Fatema, Md. Ashrafur Rahman, Maruf Hasan, Md. Abid Hossain Mollah, Md. Ismail Hosen, Noushad Karuvantevida, Ghausia Begum, Binte Zehra, Nasna Nassir, A. H. M. Nurun Nabi, K. M. Furkan Uddin, Mohammed Uddin

**Affiliations:** ^1^ Genetics and Genomic Medicine Centre, NeuroGen Healthcare, Dhaka, Bangladesh; ^2^ Department of Biochemistry and Molecular Biology, University of Dhaka, Dhaka, Bangladesh; ^3^ Department of Paediatric Neurology, Bangabandhu Sheikh Mujib Medical University, Dhaka, Bangladesh; ^4^ Department of Child Neurology, NeuroGen Healthcare, Dhaka, Bangladesh; ^5^ Department of Paediatric Neuroscience, Dhaka Shishu Hospital, Dhaka, Bangladesh; ^6^ Department of Neurology, National Institute of Neurosciences and Hospital, Dhaka, Bangladesh; ^7^ Centre for Precision Therapeutics, NeuroGen Healthcare, Dhaka, Bangladesh; ^8^ Institute of Plant Genetics, Department of Plant Biotechnology, Leibniz University Hannover, Hanover, Germany; ^9^ Department of Medicine, Dhaka Medical College, Dhaka, Bangladesh; ^10^ Department of Paediatric Neurology, National Institute of Neuroscience and Hospital, Dhaka, Bangladesh; ^11^ Department of Biology and Biochemistry, University of Houston, Houston, TX, United States; ^12^ Cellular Intelligence Lab, GenomeArc Inc, Toronto, ON, Canada; ^13^ Department of Child Neurology, Bangabandhu Sheikh Mujib Medical University, Dhaka, Bangladesh; ^14^ Department of Obstetrics and Gynaecology, Bangabandhu Sheikh Mujib Medical University, Dhaka, Bangladesh; ^15^ Department of Pharmaceutical Sciences, Wilkes University, Pennsylvania, PA, United States; ^16^ Department of Biomedical Engineering, Military Institute of Science and Technology, Dhaka, Bangladesh; ^17^ Department of Paediatrics, BIRDEM General Hospital, Dhaka, Bangladesh; ^18^ College of Medicine, Mohammed Bin Rashid University of Medicine and Health Sciences, Dubai, United Arab Emirates; ^19^ Department of Biochemistry, Holy Family Red Crescent Medical College, Dhaka, Bangladesh; ^20^ Cellular Intelligence (Ci) Lab, GenomeArc Inc, Toronto, ON, Canada

**Keywords:** neurodevelopmental disorders (NDDs), chromosomal microarray analysis (CMA), copy number variation (CNV), Autism Diagnostic Observation Schedule-Second Edition (ADOS-2), variant of uncertain significance (VOUS), critical exon gene (CEG), autism spectrum disorder (ASD)

## Abstract

**Introduction:** Copy number variations (CNVs) play a critical role in the pathogenesis of neurodevelopmental disorders (NDD) among children. In this study, we aim to identify clinically relevant CNVs, genes and their phenotypic characteristics in an ethnically underrepresented homogenous population of Bangladesh.

**Methods:** We have conducted chromosomal microarray analysis (CMA) for 212 NDD patients with male to female ratio of 2.2:1.0 to identify rare CNVs. To identify candidate genes within the rare CNVs, gene constraint metrics [i.e., “Critical-Exon Genes (CEGs)”] were applied to the population data. Autism Diagnostic Observation Schedule-Second Edition (ADOS-2) was followed in a subset of 95 NDD patients to assess the severity of autism and all statistical tests were performed using the R package.

**Results:** Of all the samples assayed, 12.26% (26/212) and 57.08% (121/212) patients carried pathogenic and variant of uncertain significance (VOUS) CNVs, respectively. While 2.83% (6/212) patients’ pathogenic CNVs were found to be located in the subtelomeric regions. Further burden test identified females are significant carriers of pathogenic CNVs compared to males (OR = 4.2; *p* = 0.0007). We have observed an increased number of Loss of heterozygosity (LOH) within cases with 23.85% (26/109) consanguineous parents. Our analyses on imprinting genes show, 36 LOH variants disrupting 69 unique imprinted genes and classified these variants as VOUS. ADOS-2 subset shows severe social communication deficit (*p* = 0.014) and overall ASD symptoms severity (*p* = 0.026) among the patients carrying duplication CNV compared to the CNV negative group. Candidate gene analysis identified 153 unique CEGs in pathogenic CNVs and 31 in VOUS. Of the unique genes, 18 genes were found to be in smaller (<1 MB) focal CNVs in our NDD cohort and we identified *PSMC3* gene as a strong candidate gene for Autism Spectrum Disorder (ASD). Moreover, we hypothesized that *KMT2B* gene duplication might be associated with intellectual disability.

**Conclusion:** Our results show the utility of CMA for precise genetic diagnosis and its integration into the diagnosis, therapy and management of NDD patients.

## Introduction

Neurodevelopmental disorders (NDDs) are a group of developmental deficits that disrupt the normal physiology and function of the brain. These disorders are referred to as a collection of early-onset developmental delay (DD) conditions that include autism spectrum disorders (ASDs), intellectual disabilities (IDs), epilepsy encephalopathy, attention-deficit hyperactive disorders (ADHDs), obsessive compulsive disorder (OCD), and cognitive skill disorders (Zoghbi, 2003; Levitt et al., 2004; Dennis et al., 2009; Bale et al., 2010; Krakowiak et al., 2012). When isolated, such disorders are termed non-syndromic, while these are referred to as syndromic when associated with dysmorphisms or apparent congenital anomalies (CAs) (Abou Jamra et al., 2011). The incidence of DD/ID is 3% in the general population (Shevell et al., 2003), while the statistics from the United States shows that ASD affects 1 in 54 live births (Knopf, 2020). Individuals affected with NDDs usually present reduced adaptive skills, limited intellectual ability, motor difficulties, CAs, and problems with social interaction. Phenotypically, there are major overlaps among ASDs with epilepsy encephalopathy, ADHD, Fragile X syndrome (FXS), motor abnormality, and intellectual disability (Matson and Shoemaker, 2009; Hampson et al., 2011).

The etiology of NDDs is principally genetic. Advancements in genomic techniques such as CMA and next-generation sequencing have yielded significant insights into the genetic etiology of NDDs (Zhang et al., 2013). For decades, structural genomic variation has been a major contributor to the etiology of a proportion of children diagnosed with NDDs (Lionel et al., 2011; Ahn et al., 2014; Liu et al., 2015b; Oskoui et al., 2015; Begum et al., 2021). In the last decade, many large international genomic consortiums have profiled NDD cases, mostly of European ancestry, to identify genomic alterations and NDD-associated genes. More than 100 genes and genomic loci (Cooper et al., 2011) have been consistently found to be involved in the etiology of NDDs. Studies based on ASD cohorts have identified an increased burden of rare genetic copy number variations (CNVs) and have characterized rare, usually *de novo*, recurrent CNV loci that contribute to genetic risk (Pinto et al., 2014). Specific genes within these CNV regions implicated in the etiology of ASD and other NDDs include *SHANK3*, *SYNGAP1*, *NRXN1*, *GRM7*, and *DLGAP2.* (Moessner et al., 2007; Hamdan et al., 2011; Chien et al., 2013; Liu et al., 2015a; Woodbury-Smith et al., 2017). As the number of candidate genes and loci has increased, a striking recurrence of candidates identified in multiple disorders has been uncovered, which may account for a proportion of the significant comorbidity that has been noted among neurodevelopmental disorders (Chen et al., 2014; Cristino et al., 2014). The availability of microarray-related technologies and the contribution of structural variations to NDD enabled CMA as one of the first-tier diagnostic tests for NDD cases in developing countries. In 2010, Miller et al. (2010) demonstrated the utility of CMA as a first-tier clinical diagnostic test to enable early diagnosis of individuals with NDDs. This technology can precisely detect 10%–30% of NDD cases (Miller et al., 2010; Ho et al., 2016; Uddin et al., 2016; Chaves et al., 2019).

In Bangladesh, child mental health is considered a significant health problem, with around 5 million Bangladeshi children and adolescents having psychiatric disorders (Mullick and Goodman, 2005). In a well-characterized NDD cohort, a gold-standard observational assessment tool ADOS-2 confirmed 73.85% (209/283) as ASD cases and the remaining 26.15% (74/283) as broader NDD cases (Rahaman et al., 2020). Here, the diagnosis of NDDs was mostly carried out by observing the clinical conditions of the patients and using psychological assessment tools (DSM-IV and ADOS/ADOS2) (Rahaman et al., 2020). Due to the overlapping and complex clinical presentation of NDDs, it is difficult to confirm NDD diagnosis by psychological assessment. Therefore, early diagnosis of NDD cases in children may lead to better outcomes through expeditious educational planning and therapeutics (Filipek et al., 1999). In Bangladesh, the genetic cause of breast cancer, intellectual disability, and rare diseases was uncovered by whole-genome sequencing, whole-exome sequencing, targeted sequencing, chromosomal microarray analysis, and quantitative PCR (Uddin K. M. F. et al., 2018; Akter et al., 2019; Rahman et al., 2019; Uddin et al., 2019; Akter et al., 2021). However, there is no comprehensive genetic study carried out with a large NDD cohort. Our study analyzed a cohort of 212 NDD patients in Bangladesh who underwent microarray testing to identify copy number variations (CNVs) from 2017 to 2020 for diagnostic purpose. To our knowledge, this is the first cohort of NDD patients reporting a significant number of clinically relevant variants and genes from the Bangladeshi population.

## Materials and methods

### Cohort description

The cohort comprised 212 neurodevelopmental disorder patients with ASD (*n* = 95), DD (*n* = 48), epilepsy/seizure (*n* = 19), ID (*n* = 15), hypotonia (*n* = 5), speech and language disabilities (*n* = 144), ADHD (13), and rare unexplained cases with syndromic features (*n* = 25). Almost all the patients have more than one phenotype (Supplementary Table S1). Of the 212 patients, 68.40% (*n* = 145) and 31.60% (*n* = 67) were men and women, respectively. Their detailed phenotype is provided in Supplementary Table S1. We collected occipitofrontal head circumference (OFC) data, measured in centimeter using a non-stretchable plastic tape, and body weight data, measured in kilogram using a calibrated weight machine. From the available data, we have shown the distribution of age, OFC, weight, and height of 202 patients (Supplementary Figures S1, S2). All patients are from the local Bangladeshi population. Around 43% patients are city-based, so they have access to resources of modern healthcare. The rest of the patients are from rural areas that lack modern healthcare resources. All these patients were referred for microarray testing after clinical evaluation by different neurologists and pediatricians from different parts of the country. From the available medical records of some patients, we have prepared a summary table keeping information such as mode of delivery, perinatal complication, percentage of premature and intrauterine dystrophic newborns, percentage of specific instrumental examinations (EEG, etc.), and ultrasonography and MRI of the CNS (Supplementary Table S2). From the available medical records, we found the birth weight of 76 patients and produced a birth weight distribution plot (Supplementary Figure S3). Moreover, a subset of patients (*n* = 95) was evaluated for autism spectrum disorders by using the ADOS-2 method. Among these 95 participants (70 men and 25 women), 71 met the criteria of autism-positive (51 men and 20 women), and the remaining 24 were classified as autism-negative. In addition to the 71 patients , 24 more autism patients were diagnosed by using different assessment tools, including the DSM-V (*n* = 14) and ADOS (*n* = 10). Therefore, in total, there were 95 ASD patients (44.81% = 95/212) diagnosed using different psychological assessment tools, DSM-V (*n* = 14), ADOS (*n* = 10), and ADOS-2 (*n* = 71), with a male-to-female ratio of 2.65:1.00 and age ranging from 1.5 years to 19 years (Supplementary Table S1).

### Autism assessment

Autism Diagnostic Observation Schedule-Second Edition (ADOS-2) (Lord et al., 2012) is a semi-structured, standardized assessment of communication, social interaction, imaginative use of materials, and restricted and repetitive behaviors. ADOS-2 is a gold-standard observational assessment tool for diagnosing ASD. ADOS-2 has five modules, and each module offers standard activities designed to elicit behaviors relevant to diagnosing ASD at different chronological ages and language abilities. In this study, autism characteristics were measured using the Toddler module, Module 1, and Module 2. Each module uses communication, reciprocal social interaction, and restricted and repetitive behaviors to generate a total score. Elevated scores classify an individual in the autism spectrum or autism diagnostic range based on the severity or the frequency displayed.

### DNA extraction and quantitation

DNA was extracted from peripheral blood samples using the ReliaPrep™ Blood gDNA isolation kit (Promega, United States) following the manufacturer’s protocol. Extracted DNA samples were checked for quality using a NanoDrop spectrophotometer. Samples were electrophoresed on agarose gels, and samples with intact genomic DNA showing no smearing on agarose gel were selected for the experiment. Intact genomic DNA was diluted to 50 ng/μL concentration based on Quant-iTPicoGreen (Invitrogen) quantitation. The whole-genome amplification process requires 200 ng of input gDNA.

### Chromosomal microarray analysis

We conducted CMA to identify copy number variations and investigated the changes in fluorescence intensities between the test specimen and the controls. Illumina Global Screening Array-24 + v1.0 was used applying Illumina SNP genotyping technology and Illumina CNVPartition 3.2.1 plug-in of GenomeStudio to detect chromosomal abnormalities using the reference genome GRCh38/hg38. This microarray uses 642,824 probes spread across the genome to detect genetic abnormalities (includes >60 loci in the DECIPHER database reported for neurodevelopmental disorders) greater than 30 kb (for deletion and duplication CNV) and targets sub-telomeric regions vulnerable to chromosomal abnormalities. We analyzed loss of heterozygosity (LOH) within a 2–10 MB range for further analysis due to low-resolution array and false-positive calls. We used rigorous multiple algorithmic techniques (MatLab and Java) and manual curation of the data to pinpoint genomic variation based on the normalized log2 intensities of the probes. Our algorithm excluded all common CNVs found in the in-house (NeuroGen) control population samples (9,689 samples) from analysis and only rare (frequency <0.01) CNVs to infer their contribution to human diseases. Sample digestion, ligation, PCR, labeling, hybridization, and scanning were performed following the standard protocols.

### Frequency determination

We used a commercially available tool, GenomeArc (GenomeArc Inc.), an annotation software that integrates clinical, genomics, and OMICs data. To determine the frequency, >50% reciprocal overlap within the same chromosome was considered, and we used 9,689 unrelated population control samples from European ancestry (Uddin et al., 2016). These samples were collected from multiple major population-scale studies that used high-resolution microarray platforms. These included 4,347 control samples assayed by Illumina 1 M from the Study of Addiction Genetics and Environment (SAGE) (Bierut et al., 2010) and the Health, Aging, and Body Composition (HABC) (Coviello et al., 2012); 2,988 control samples were assayed by Illumina Omni 2.5 M from the Collaborative Genetic Study of Nicotine Dependence (COGEND) (Bierut et al., 2007) and Cooperative Health Research in the Region of Augsburg KORA projects (Verhoeven et al., 2013); and 2,357 control samples were assayed by Affymetrix 6.0 from the Ottawa Heart Institute (Stewart et al., 2009) and the PopGen project (Krawczak et al., 2006).

### CNV classification criteria

To classify and assess the clinical relevance of a particular CNV, at first, all common CNVs (frequency >1%) found in the control population samples (9,689 samples) and Database of Genomic Variants (DGV) (http://dgv.tcag.ca/dgv/app/home) were excluded from further analysis. Then, rare CNVs (frequency<1%) were classified based on their type (gain or loss or LOH), size, location, gene content, and patients’ clinical data. By searching genomic databases, including Online Mendelian Inheritance in Man (OMIM) (https://omim.org/), ClinVar (https://www.ncbi.nlm.nih.gov/clinvar/), ClinGen Dosage Sensitivity Map (https://dosage.clinicalgenome.org), ISCA database (http://dbsearch.clinicalgenome.org/search/), DECIPHER (https://decipher.sanger.ac.uk/), and published literature (http://www.ncbi.nlm.nih.gov/pubmed/), CNVs were finally classified as pathogenic, variants of uncertain significance (VOUS), and benign following the ACMG guidelines (Kearney et al., 2011).

### Droplet digital PCR

Copy number assays were performed using the Droplet Digital PCR (ddPCR) System (Bio-Rad Laboratories, Inc.). The GeneAssist™ Copy Number Assay Workflow Builder (Thermo Fischer, United States) was used to design TaqMan assays on Chr11, *PKNOX2* (Hs03289418_cn), Chr15, *SNHG14* (Hs05375107_cn), and Chr21, *TSPEAR* (Hs02835328_cn) with FAM dye. TaqMan™ Copy Number Reference Assay, human, RNase P with VIC dye was used as a reference assay. A total of 22 µL reaction mix was prepared, containing 3.5 µL of template DNA (20 ng/μL) without restriction digestion, 10 µL of 2X ddPCR supermix for probes (No UDP) (Bio-RAD Laboratories Inc.), 1 µL each 20X TaqMan target probe (FAM) and 20X TaqMan reference probe (VIC) (Applied Biosystems, United States), and 6.5 µL of RNase-/DNase-free water. All reactions were prepared in triplicates with one negative control. The reaction mixtures were partitioned using the QX200 Droplet Generator™ and then transferred to a 96-well plate and amplified using the C1000 Touch thermal cycler, as per the manufacturer’s protocol. Then, the samples were read using the QX200 Droplet Reader™. Data acquisition and analysis were performed using QuantaSoft Version 1.7.4.0917, and the Poisson algorithm was used to determine the concentrations of the targets as copies/μL.

### Candidate gene and pathway analysis

We used pathogenic CNVs for enrichment analysis (https://github.com/MBRULab/2021_Nassir-etal/blob/main/geneoverlap.R) and mapping. We scanned the KEGG pathway database, which comprises an assembly of the up-to-date interactions, reactions, and relations of molecular networks (https://www.genome.jp/kegg/pathway.html), and the GO database (http://geneontology.org/) to identify all the pathways in which five or more genes were expressed. Only the pathways having more than 50 genes and less than 1,000 genes were considered for this analysis (Cline et al., 2007; Bader et al., 2014; Nassir et al., 2021). The pathways were identified by their unique KEGG ID and name. A pathway was considered significant if the false discovery rate (FDR) and *p*-value cut-off were <0.01 and 0.001, respectively. Then, the network was built using the enrichment map and the auto-annotate Cytoscape (https://cytoscape.org/) application. *P*-values are denoted using a color gradient (low *p-*values with darker colors). Furthermore, the “critical-exon” method (Uddin et al., 2014; Uddin et al., 2016) was applied to identify candidate genes within the pathogenic and VOUS breakpoints. “Critical exons” are highly expressed in the brain and have low population rare mutation burden. Genes with critical exons are called critical exon genes.

### Statistical analysis

Welch’s *t*-test was used to determine the significant difference between the means of the two groups. A significance level of 0.05 was determined to test differences. For qualitative data, Fisher’s exact test was applied. All analyses were performed using the R package.

## Results

The cohort comprised 212 neurodevelopmental disorder patients with a male-to-female ratio of 2.2:1.0 (Supplementary Table S1). In this cohort, 95 patients were evaluated for autism spectrum disorder using the ADOS-2 method, and 71 met the ADOS-2 cut-off criteria of autism-positive (51 men and 20 women, male/female ratio 2.55:1.00), while the remaining 24 were classified as autism-negative (non-spectrum). We observed that patients carrying duplication CNVs showed severe social communication deficit (*p* = 0.014) and overall ASD symptom severity (*p* = 0.026) compared to the CNV-negative group (patients with no clinically rare variants). Moreover, a trend of increased number of CNVs in autism patients was observed in comparison to non-spectrum individuals (OR = 2.29; *p* = 0.06).

**FIGURE 1:**
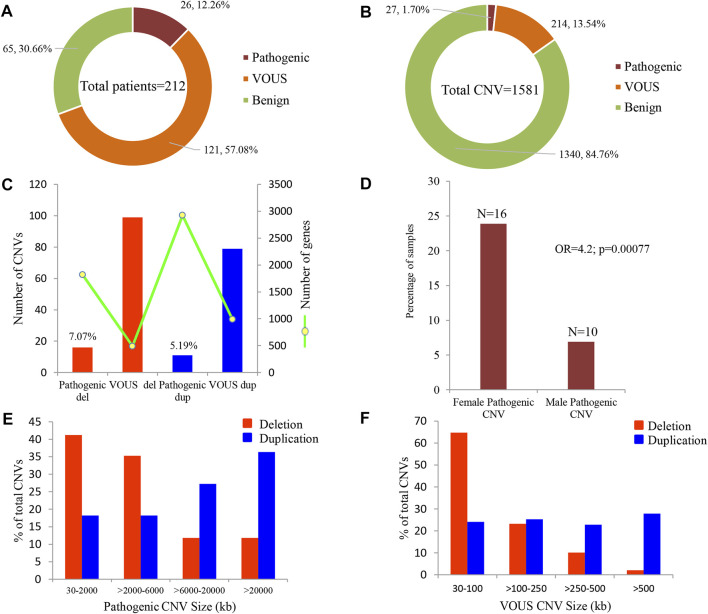
Summary of CNVs identified in 212 NDD patients. **(A)** Percentage of patients carrying pathogenic, VOUS, and benign CNVs. Of all samples assayed, 12.26% patients carried pathogenic CNVs, where 7.07% carried a pathogenic deletion and 5.19% a pathogenic duplication. **(B)** Percentage of pathogenic, VOUS, and benign CNVs. Of the total 1,581 CNVs, 1.7% are pathogenic, 13.54% are VOUS, and the remaining 84.76% are benign. **(C)** Bars indicate the total number of CNVs in deletion and duplication. The green line represents the number of unique genes impacted by the corresponding variants. **(D)** Percentage of male and female groups in the full cohort impacted by pathogenic CNVs. *p*-value was calculated by Fisher’s exact test. **(E)** and **(F)** size distribution of pathogenic and VOUS CNVs. The average length of pathogenic deletion and duplication is 5365.26 kb and 17,451.72 kb, respectively, whereas the average length of VOUS deletion and duplication is 129.17 kb and 476.76 kb, respectively.

**FIGURE 2:**
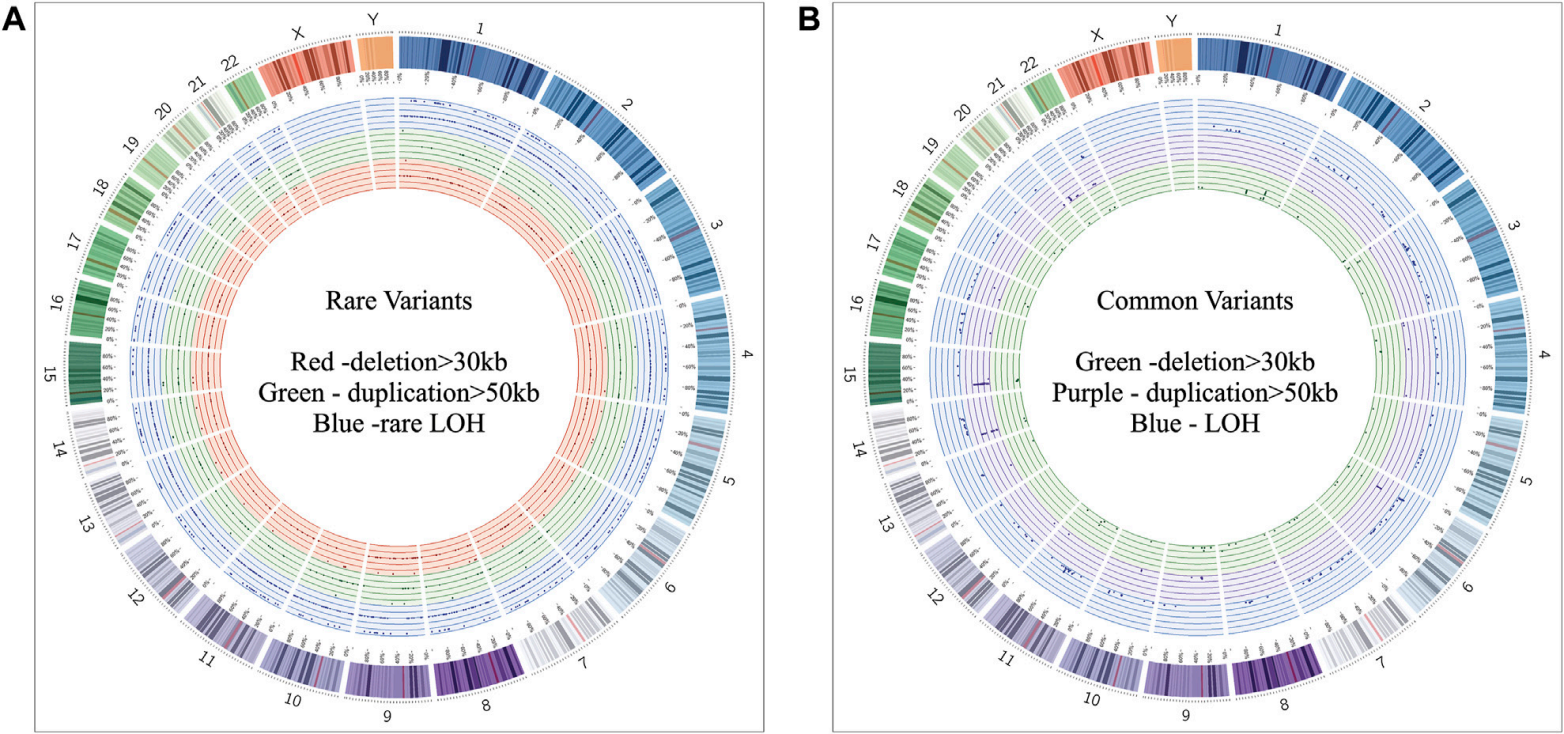
Circos (Krzywinski et al., 2009) plot illustrating neurodevelopmental disorder map showing the frequency of rare and common CNVs. **(A)** Frequency distribution of all rare CNVs throughout chromosomes 1 to 22. Dots in the red, green, and blue circles indicate frequency of >30 kb deletion, >50 kb duplication, and LOH CNVs, respectively. Within a colored circle, the outermost sub-circle contains the maximum value of 0.01, and the inner sub-circles contain values less than 0.01 but more than 0.001. **(B)** Frequency distribution of all common CNVs throughout chromosomes 1 to 22. Dots in the green, purple, and blue circles indicate frequency of >30 kb deletion, >50 kb duplication, and LOH CNVs, respectively. Each colored circle contains frequency values more than 0.01.

Of all samples assayed, 12.26% (26/212) and 57.08% (121/212) patients carried pathogenic and variant of uncertain significance (VOUS) CNVs, respectively (Figure 1A). We identified 1,581 CNVs (excluding <30 kb deletion,<50 kb duplication, <2 Mb, and >10 Mb LOH) that included 395 duplications, 658 deletions, and 528 LOH interpreted and classified according to the ACMG guidelines (Kearney et al., 2011) into pathogenic (*n* = 27), variant of uncertain clinical significance (VOUS) (*n* = 214) and benign (*n* = 1,340) CNVs (Figure 1B). Of the 27 pathogenic CNVs, recurrent deletions and duplications were identified in the 15q11.2q13.1 and 21q11.1q22.3 loci (Table 1). In this cohort, 2.83% (6/212) and 20.28% (43/212) patients carried pathogenic and VOUS subtelomeric CNVs, respectively (Supplementary Table S1). Of the 26 samples carrying pathogenic CNVs, one patient was carrying double terminal pathogenic deletion impacting chromosome 18 (Table 1), and 11 patients with a pathogenic CNV were also carrying VOUS CNV (Supplementary Table S3). The 27 pathogenic CNVs comprised 16 deletions and 11 duplications (Table 1; Figure 1C). It was observed that women are the more substantial carriers of pathogenic CNVs than men (OR = 4.2; *p* = 0.0007) (Figure 1D). The average length of deletion and duplication was 5365.26 kb and 17,451.72 kb, respectively, and the highest frequency group for pathogenic CNV deletion and duplication was 30–2000 kb and >20000 kb, respectively (Figure 1E). To exclude false CNV calls, we randomly chose nine pathogenic/likely pathogenic CNVs for ddPCR validation, and it yielded an (8/9) 88.89% validation rate (Supplementary Figure S4, Supplementary Table S4). In this validation, a single 775.77 kb deletion variant (#case 7) did not show accurate log intensity data due to the algorithm’s false call on the low resolution of the microarray. A large number of genes were found to be disrupted in pathogenic deletion (*n* = 1846) and duplication CNVs (*n* = 2,925) compared to VOUS deletions (*n* = 490) and duplications (*n* = 991) (Figure 1C). The average length of VOUS deletion and duplication was 129.17kb and 476.76 kb, respectively, and the highest frequency group for VOUS CNV deletion and duplication was 30–100 kb and >500 kb, respectively (Figure 1F). Furthermore, we determined the frequency of all 1,581 deletion, duplication, and LOH CNVs within 212 Bangladeshi NDD patients and 9,689 control CNVs from the Ontario population, which is predominantly European in ancestry. Using the frequency distribution of both rare and common CNVs of NDD patients from Bangladesh, a Circos map was constructed (Krzywinski et al., 2009) (Figure 2). Consanguinity increases the possibility of inheriting recessive diseases, and in our Bangladeshi cohort, 23.85% (26/109) families were found to be consanguineous. In the children born to consanguineous parents, we found an increased number (12.95 LOH/patient) and larger size (>6 Mb) of LOH compared to that found in the children of non-consanguineous parents (Supplementary Figures S5, S6). The pathogenesis of LOH includes homozygous mutation of recessive diseases and imprinting effects caused by uniparental disomy (UPD). However, without whole-genome sequencing data of the genes associated with recessive diseases, it is difficult to address the severity of LOH-harboring recessive genes. We have thoroughly checked the LOH for imprinted genes and found 36 LOH-disrupting 69 unique imprinted genes and classified these variants as VOUS (Supplementary Table S5).

**TABLE 1:** Details of pathogenic CNVs found in the cohort.

ID	Sex	Type	Chromosomal band and position (hg38)	Size (Mb)	Number of affected genes	Critical exon genes	Known syndrome/genes associated with developmental disorder within the breakpoints	Clinical conditions	BD frequency (*n* = 212)	Canada control frequency (*n* = 9,692)
1	M	Del^a^	4p16.3p16.2 chr4:35,031-4,878,160	4.843	155	*CTBP1*, *PCGF3*, *FAM193A*, and *HTT*	Wolf–Hirschhorn Syndrome; *PIGG*, *UVSSA3*, *IDUA*, *FGFR3*, *LRPAP1*, *SH3BP2*, and *MSX1*	Autism, developmental delay (walking at 5 years), behavioral problem, intellectual disability, frequent falling, and dysmorphic face	0.00472	0
19	M	Del^b^	15q11.2q13.1 chr15:22,838,642-28,314,382	5.476	170	*GABRB3*, *CYFIP1*, *UBE3A*, *HERC2*, and *GABRA5*	PWS/AS; *UBE3A*, *GABRB3*, and *MAGEL2*	Global developmental delay, cannot stand spontaneously, fatty body, and syndromic child	0.01415	0
20	F	Del	20p12.3 chr20:8,117,650-8,593,664	0.476	1	*PLCB1*	*PLCB1*	Autism, speech delay, temper tantrum, and restlessness	0.00472	1.03E-04
35	F	Del^b^	15q11.2q13.1 chr15:22,777,709-28,736,935	5.959	181	*GABRB3*, *CYFIP1*, *UBE3A*, *HERC2*, and *GABRA5*	PWS/AS; *UBE3A; GABRB3;* and *MAGEL2*	Hypotonic	0.01415	0
40	F	Del	17p11.2 chr17:16,858,825-20,387,706	3.529	136	*LLGL1*, *SREBF1*, *COPS3*, and *AKAP10*	Smith–Magenis syndrome; Potocki–Lupski_syndrome_(17p11.2_duplication_syndrome); *TNFRSF13B*, *RAI1*, *B9D1*, and *ALDH3A2*	Behavioral problem, speech delay, and low hairline	0.00472	0
53	F	Del	1q24.2q31.2 chr1:168,503,684-191,056,769	22.553	360	*TPR*, *NMNAT2*, *DHX9*, *RGL1*, *SMG7*, *PRRC2C*, *GLUL*, *CACNA1E*, *IVNS1ABP*, *RNF2*, *ASTN1*, *CACYBP*, *ATP1B1*, *VAMP4*, *TNR*, and *XPR1*	*GORAB*, *TSEN15, LHX4*, *ACBD6*, *GLUL*, *PRRX1*, *NPHS2*, *DARS2*, and *MYOC*	Global developmental delay, high ammonia and lactate levels, and EEG-normal	0.00472	0
59	M	Del	17q12 chr17:36,201,251-37,889,808	1.689	51	*GGNBP2* and *ACACA*	RCAD (renal_cysts_and_diabetes); *HNF1B, PIGW*	Global developmental delay (delayed walking and speech), salivation, restlessness, and H/O pneumonia at 15 days of age	0.00472	0
86	M	Del^a^	18p11.32 chr18:45,680-2,276,243	2.231	38	*COLEC12*	Null	Developmental delay and complete cleft lip with cleft palate	0.00472	0
			18q21.31q23 chr18:58,298,202-80,227,529	21.929	280	*PHLPP1*, *KIAA1468, NEDD4L*, *ZNF532*, *NETO1*, and *TSHZ1*	*CTDP1, RTTN, NEDD4L, TSHZ1, PIGN, TXNL4A, RAX, CCBE1,* and *CTDP1*		0.00472	0
87	F	Del	8q22.3 chr8:103,458,677-103,560,657	0.102	RIMS2	Null	*RIMS2*	Autism, impaired language development, restlessness and hyperactivity, and delayed walking	0.00472	0
107	F	Del	7q11.23 chr7:73,294,506-74,715,504	1.421	40	*BAZ1B, LIMK1, STX1A, EIF4H,* and *CLIP2*	Williams–Beuren syndrome (WBS); 7q11.23 duplication syndrome; *ELN*	Developmental delay (unable to sit without support), feeding problem, jerky movement, and hypotonic	0.00472	0
144	F	Del^a^	2q37.1q37.3 chr2:233,505,099-242,065,217	8.560	219	*KIF1A, ATG4B, AGAP1, UBE2F,* and *HDLBP*	2q37 monosomy; *KIF1A, COL6A3, NDUFA10, D2HGDH, CAPN10, TWIST2, HDAC4, UGT1A1,* and *AGXT*	Syndromic child, developmental delay, unable to sit without support, poor neck control, no speech, sleeping problem at night, and lack of social response. Eyes closed for 2 days after birth, breathing with oxygen support and needed blood transfusion. Pneumonia occurred on day 28	0.00472	0
159	M	Del	2q24.1 chr2:154,077,277-158,596,423	4.519	70	*KCNJ3, NR4A2,* and *ACVR1*	*ACVR1*	Intellectual disability, ADHD, speech delay, involuntary movement, abnormal behavior, self-injurious behavior, and aggressive behavior. History of epilepsy two times. At 6 months of age, patient suffered from pneumonia. Facial appearance: normal	0.00472	0
178	M	Del	15q11.2q13.1 chr15:22,600,363-28,760,485	6.160	188	*GABRB3, CYFIP1, UBE3A, HERC2,* and *GABRA5*	PWS/AS; *UBE3A, GABRB3,* and *MAGEL2*	Spastic quadriplegia/CP, global developmental delay, speech problem, and cannot sit without support. CT scan of the brain: atrophy ventricular dilation. Dysmorphic face	0.01415	0
182	F	Del	2q24.3 chr2:165,992,388-166,079,596	0.087	1	Null	*SCN1A*	Global developmental delay (poor neck control, cannot sit or walk, and speech delay), cerebral palsy, epilepsy starts at the age of 5 months, history of frequent infantile spasm, flexor spasm, sleeping problem, eating problem (only liquid food), restlessness, crying nonstop, and drooling of saliva. Her elder brother deceased at the age of 8 months after suffering from pneumonia. He also had delayed development; his neck was also weak, anemic during pregnancy. Facial appearance: abnormal	0.00472	0
184	M	Del	17q12 chr17:34,815,552-36,249,430	1.434	76	*AP2B1*	*PEX12*	Low-level autism, epilepsy, ADHD, club foot, temper tantrum, speech delay, self-smiling, aggressive, poor response, and poor peer relationship. Admitted to the NICU at the age of 4 days due to convulsion for 2 days, and re-hospitalized at 15 days of age due to cold attack and sleeping problem. Family history: paternal cousin was hyperactive and father had seizure during childhood	0.00472	0
5	F	Dup^a^	3p26.3p26.2 chr3:2,299,060-3,322,758	1.024	5	null	*CRBN*	Expressive language disorder	0.00472	0
77	F	Dup	9p24.3p12 chr9:48,828-39,297,860	39.249	619	*PTPRD, MLLT3, SH3GL2, CDC37L1, SMARCA2, NOL6, KLHL9, NFIB, PSIP1, SMU1, ELAVL2, UBAP1, RUSC2, UBE2R2, CLTA, CNTFR, VCP, NPR2, TESK1, TLN1,* and *RNF38*	*PIGO, IL11RA, NPR2, TPM2, GBA2, GALT, FANCG, EXOSC3, RMRP, MPDZ, GLIS3, DDX58, VLDLR, KANK1, APTX, FREM1, DOCK8, TEK; SMARCA2, GLIS3, PLAA, TYRP1,* and *GLDC*	Developmental delay, speech delay, intellectual disability, and anorexia	0.00472	0
14	M	Dup^b^	11q23.2q24.2 chr11:114,071,597-125,513,912	11.442	307	*BCL9L, BACE1, IFT46, DDX6, C2CD2L, GRAMD1B, UBE4A, CADM1, HSPA8, HYOU1, ARHGEF12, SIK3, ARCN1, PKNOX2,* and *PAFAH1B2*	*ZBTB16, NECTIN1, ARCN1, ROBO3, DPAGT1, SC5D, CLMP, CBL, KMT2A,* and *MFRP*	Developmental delay (speech delay), poor eye contact, history of seizure, cerebral palsy, facial dysmorphism present, and corpus callosum agenesis	0.00472	0
15	F	Dup^a^	14q32.11q32.33 chr14:90,576,836-106,816,816	16.240	626	*DICER1, ITPK1, TTC7B, TRAF3, PAPOLA, TECPR2, AKT1, DYNC1H1, EVL, WARS, YY1, CDC42BPB, BCL11B, PPP2R5C, INF2, EIF5, PACS2,* and *MTA1*	*TRIP11, UBR7, SLC24A4, CCDC88C, APOPT1, TECPR2, VRK1, DYNC1H1, AKT1, YY1,* and *PACS2*	High level of salivation, lack of self-care, and developmental delay	0.00472	0
2	M	Dup^b^	15q11.2q13.1 chr15:23,443,797-28,289,373	4.846	147	*GABRB3, UBE3A, HERC2,* and *GABRA5*	PWS/AS; *UBE3A, GABRB3,* and *MAGEL2*	Autism, syndromic child	0.009434	0
101	F	Dup^b^	15q11.2q13.1 chr15:23,370,969-28,371,148	5.000	157	*GABRB3, UBE3A, HERC2,* and *GABRA5*	PWS/AS; *UBE3A, GABRB3,* and *MAGEL2*	Autism, impaired language development, abnormal behavior, stereotypical hand movement, restrictive and repetitive activity, restlessness and hyperactivity, and unable to play with peers. High lactic acid and ammonia levels. Facial appearance: normal	0.009434	0
79	M	Dup^a^	17p13.3p12 chr17:151,597-11,392,231	11.241	414	*PRPF8, PITPNA, ABR, YWHAE, MNT, SMG6, TNFSF12, SENP3, RAP1GAP2, CHD3, CAMTA2, KDM6B, DLG4, NLGN2, ANKFY1, PHF23, FXR2, POLR2A, MINK1, ZBTB4, CTDNEP1, RABEP1, TNFSF12-TNFSF13, EIF4A1, PAFAH1B1,PITPNM3, NEURL4, RPL26, MYH10,* and *NDEL1*	Miller–Dieker syndrome (MDS); *INPP5K, KANSL1, WDR81, BHLHA9, WRAP53, DLG4, TRPV3, ACADVL, ASPA, C1QBP, MPDU1, KDM6B, SLC13A5, CTNS, AIPL1, AIPL1, MYH10, CTC1, MYH3, MYH8,* and *SCO1*	Speech delay, hyperactivity, restlessness, behavioral problem, history of seizure, seizure-free for >5 years, sleeping problem, and presence of facial dysmorphism	0.00472	0
134	F	Dup^b^	21q11.1q22.3 chr21:12,987,574-46,679,698	33.692	683	*NRIP1, ETS2, COL6A1, ITSN1, PDXK, CCT8, SON, AGPAT3, NCAM2, BRWD1, DYRK1A,* and *TIAM1*	Down’s syndrome; *C21orf59, SON, HLCS, KCNE1, DYRK1A, C21orf2, COL18A1, PCNT, RSPH1, RIPK4, CBS, FTCD, SIK1, COL18A1, CRYAA, AIRE,* and *CSTB*	Global developmental delay, feeding problem, no toilet training, speech delay, vomiting tendency, hyperactivity, and facial dysmorphism present (flat nose, Down’s syndrome -like eyes and body)	0.01415	0
136	F	Dup^b^	21q11.1q22.3 chr21:12,987,574-46,679,698	33.692	683	*NRIP1, ETS2, COL6A1, ITSN1, PDXK, CCT8, SON, AGPAT3, NCAM2, BRWD1, DYRK1A,* and *TIAM1*	Down’s syndrome; *C21orf59, SON, HLCS, KCNE1, DYRK1A, C21orf2, COL18A1, PCNT, RSPH1, RIPK4, CBS, FTCD, SIK1, COL18A1, CRYAA, AIRE,* and *CSTB*	Developmental delay, dysmorphic facial appearance, nystagmus, speech delay, low eye contact, and eating problem. Family history: low IQ in the mother	0.01415	0
152	F	Dup^b^	21q11.1q22.3 chr21:12,987,574-46,679,698	33.692	683	*NRIP1, ETS2, COL6A1, ITSN1, PDXK, CCT8, SON, AGPAT3, NCAM2, BRWD1, DYRK1A,* and *TIAM1*	Down’s syndrome; *C21orf59, SON, HLCS, KCNE1, DYRK1A, C21orf2, COL18A1, PCNT, RSPH1, RIPK4, CBS, FTCD, SIK1, COL18A1, CRYAA, AIRE,* and *CSTB*	Developmental delay and ventricular septal defect (VSD) surgery. He had CHD in the early neonatal period and was diagnosed as having large aortic VSD with inlet extension, large PDA, mild TR, and severe pulmonary hypertension. He had a history of fast breathing, feeding difficulty with failure to thrive, excessive sweating, and repeated LRTIs since the neonatal period	0.01415	0
6	F	Dup	22q11.22q11.23 chr22:22,749,561-24,600,663	1.851	114	*BCR, SMARCB1,* and *SPECC1L*	22q11.2 distal deletion syndrome; *SPECC1L* and *SMARCB1*	Restlessness, low memory, and a tendency of tearing body clothes. CT scan: normal	0.00472	1.03E-04

^a^ Subtelomeric CNV; CNV, copy number variation; CP, cerebral palsy; BD, Bangladesh; del, deletion; dup, duplication; F, female; M, male.

^b^ Each CNV validated by droplet digital PCR (ddPCR) with TaqMan assay.

**FIGURE 3:**
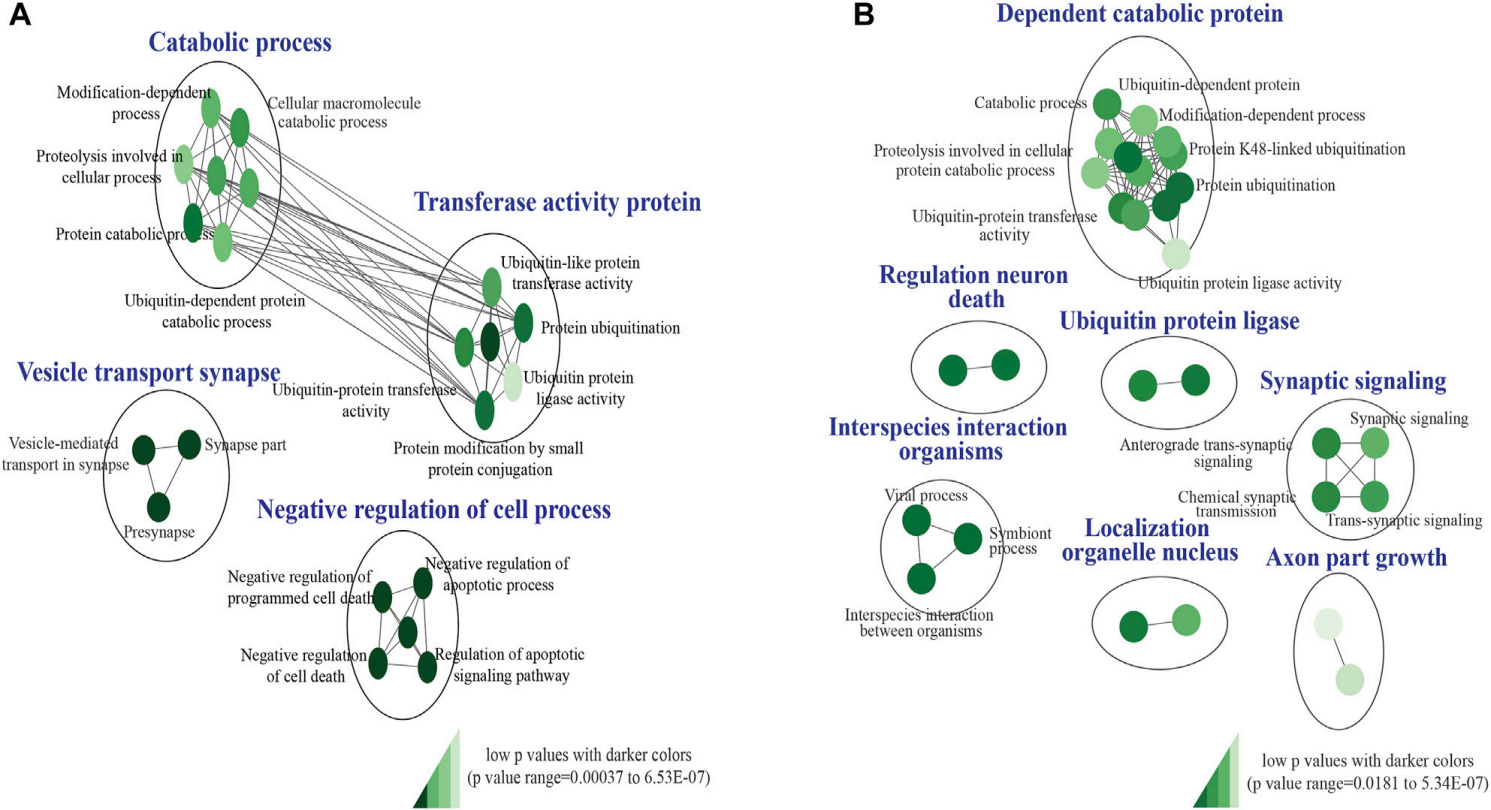
KEGG and Gene ontology pathway enrichment analysis of pathogenic CNVs identified in the study cohort. **(A)** Impacted genes within the CNV breakpoints of all pathogenic deletions identified “GO:0019787,” “GO:0060548,” and “GO:0099003” pathways to be highly significant. **(B)** Impacted genes within the CNV breakpoints of all pathogenic duplications identified “GO:0044419,” “GO:0030163,” “GO:1901214,” and “GO:0099536” pathways to be highly significant. The false discovery rate (FDR) and *p*-value cut-off were 0.01 and 0.001, respectively. *P*-values are denoted using a color gradient (low *p-*values with darker colors).

Our custom pathway enrichment (comprising Gene Ontology and KEGG databases) analysis of the impacted genes within the CNV breakpoints of all pathogenic deletions identified “ubiquitin-like protein transferase activity (GO:0019787),” “negative regulation of cell death (GO:0060548),” and “vesicle-mediated transport in synapse (GO:0099003)” pathways to be highly significant (FDR *p* < 6.53 × 10^−7^, FDR *p* < 7.2 × 10^−6^, and FDR *p* < 7.1 × 10^−5^) after correction for multiple tests (Figure 3A). Pathway enrichment on the genes impacted by pathogenic duplications identified “interspecies interaction between organisms (GO:0044419),” “dependent protein catabolic process (GO:0030163),” “regulation of neuronal death (GO:1901214),” and “synaptic signaling (GO:0099536)” pathways to be highly significant (FDR *p* < 1.51 × 10^−5^, *p* < 1.85 × 10^−5^, *p* < 5.34 × 10^−7^, and *p* < 1.72 × 10^−4^) after correction for multiple tests (Figure 3B).

**FIGURE 4:**
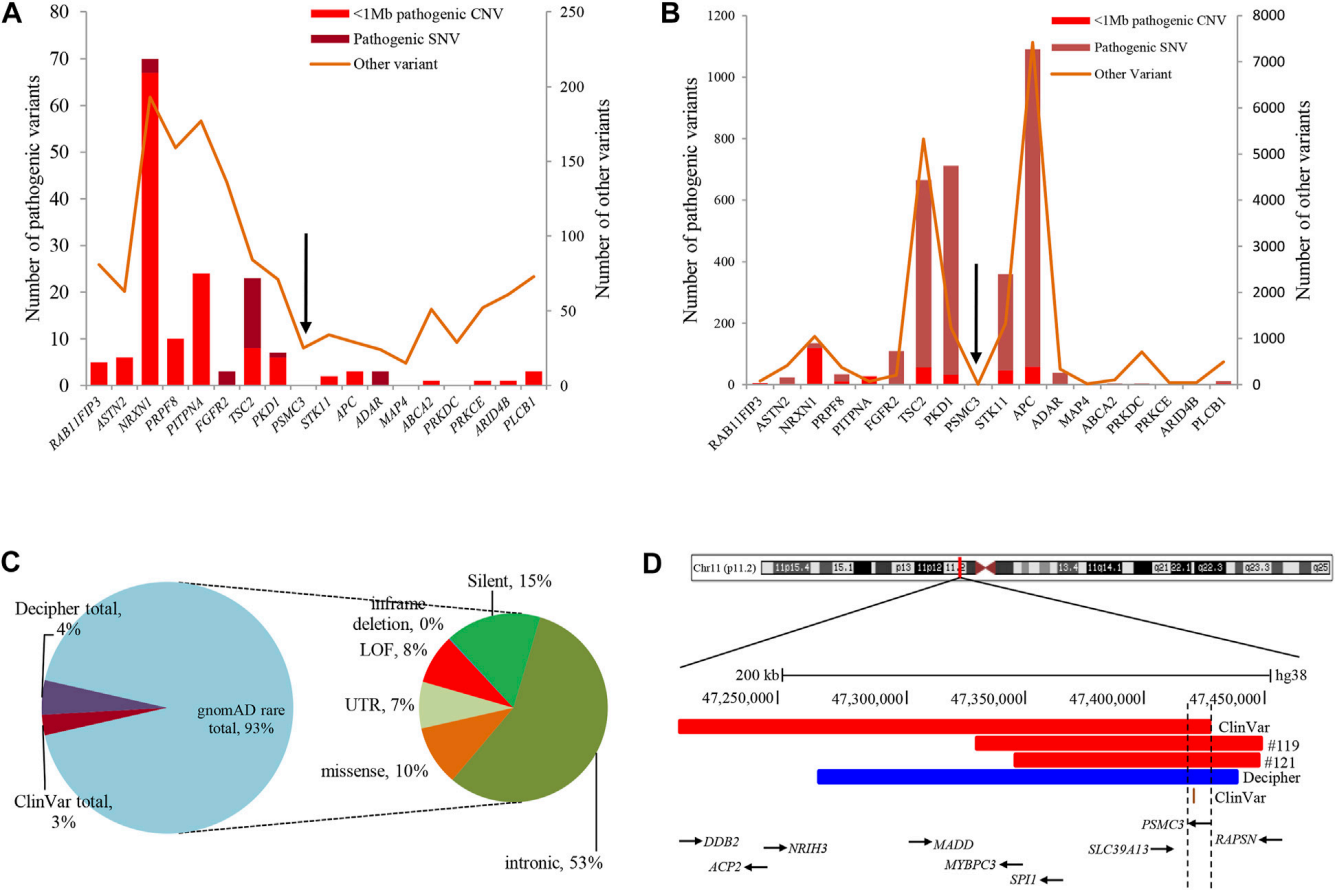
Summary of variants in 18 CEGs from various databases. **(A)** and **(B)** List of variants present in 18 CEGs from DECIPHER (Bragin et al., 2014) and ClinVar (Landrum et al., 2016) databases, respectively. **(C)** Summary of 529 rare SNVs in the *PSMC3* gene from the gnomAD (Karczewski and Francioli, 2017) database. **(D)** Schematic representation of the overlapping CNVs in our patients (#119 and #121) and previously reported cases. A close view of chromosome band 11p11.2 is displayed on the top. The comparison of the deleted regions in our patients with the previously reported CNVs identified a minimum overlapping critical region of 7.7 kb (chr11:47,418,769-47,426,473) disrupting *PSMC3*, in common. Red and blue rectangles symbolize *PSMC3*-involving gross deletion and duplication, respectively, and the orange single bar symbolizes *PSMC3*-involving SNP. CEG, critical exon gene; SNV, short-nucleotide variation; SNP, single-nucleotide polymorphism; gnomAD = The Genome Aggregation Database.

**FIGURE 5:**
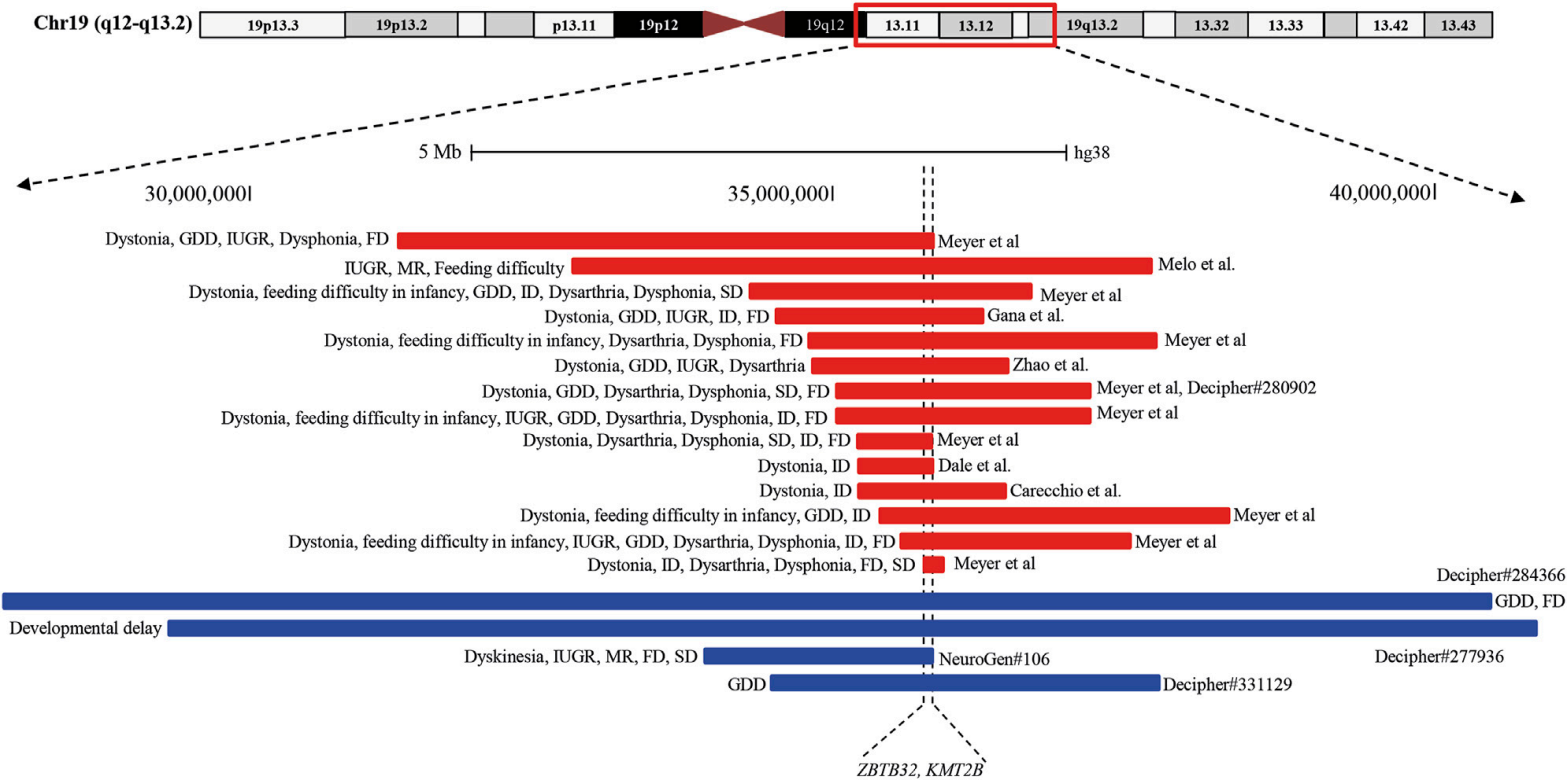
Schematic representation of the overlapping CNVs in our patient (#106) and previously reported cases. A close view of chromosome band 19q12-q13.2 is displayed on the top. The comparison of the duplicated region in our patients with the previously reported CNVs identified a minimum overlapping critical region of 38.4 kb (chr19:35,700,296-35,738,700), disrupting two genes, *ZBTB32* and *KMT2B*, in common. Red and blue rectangles symbolize *KMT2B*-involving gross deletion and duplication, respectively.

Analysis of both pathogenic and VOUS CNVs for critical exon genes (CEGs) yielded 153 unique CEGs in pathogenic CNVs and 31 in VOUS, including three genes found in both pathogenic CNVs and VOUS. On average, there were significantly more CEGs per pathogenic variant (7.5) than those in VOUS (0.19) (*p* = 0.0002) (Table 2). Through “critical-exon” analysis, we found 24 focal CNVs of <1 Mb that disrupt 22 unique CEGs in 22 NDD patients. We excluded four CEG genes: *PTPRD*, *RBFOX1*, *PCDH9*, and *LRRC4C* from further analysis, as only the intronic regions of the genes were disrupted by corresponding CNVs. After excluding these genes, the remaining 18 genes (*RAB11FIP3, ASTN2*, *NRXN1*, *PRPF8*, *PITPNA, FGFR2*, *TSC2*, *PKD1*, *PSMC3*, *STK11*, *APC*, *ADAR*, *MAP4*, *ABCA2*, *PRKDC*, *PRKCE*, *ARID4B*, and *PLCB1*) were analyzed further (Supplementary Table S6; Figures 4A, B). Of the 18 genes, we found the *PSMC3* gene as a possible candidate for ASD (Supplementary Table S7; Figure 4). We also found that a patient (#106) carrying 2.01 Mb duplication CNVs had the most similar phenotypes to the *KMT2B-*related disorder patients previously described (Zech et al., 2016; Meyer et al., 2017; Zech et al., 2017; Faundes et al., 2018; Zech et al., 2019; Cif et al., 2020) (Figure 5; Supplementary Table S8).

**TABLE 2:** Critical exon genes (CEGs) identified in CNVs from neurodevelopmental disorder.

	Pathogenic	VOUS
Total CNVs	26	178
Total number of CEGs identified	195	33
Unique CEGs identified	153	31
Average number of CEG/CNV	7.50	0.19
*t (p)	4.33 (0.0002)	

*Welch’s *t*-test.

## Discussion

This is the first Bangladeshi NDD-cohort-conducted CMA analysis. Using the GenomeArc Analytics tool, we detected a diagnostic yield of 12.26% (26/212), which is in strong agreement with other ethnically diverse neurodevelopmental disorder cohorts (Miller et al., 2010; Uddin et al., 2016; Chaves et al., 2019). Of the 12.26% (26/212), five patients were carrying different lengths of deletion and duplication CNVs in the 15q11.2q13.1 region of chromosome 15 (Table 1; Supplementary Figure S7). The proximal long arm ‘q’ of human chromosome 15q11-q13 harbors a cluster of imprinted genes, the expressions of which are controlled by the imprinting center (Buiting et al., 1995). Disruptions of this region due to deletion or duplication CNVs are associated with three distinct neurodevelopmental disorders: Prader–Willi syndrome (PWS, #176270), Angelman syndrome (AS, #105830), and 15q11-q13 duplication syndrome (Dup15q syndrome, #608636) (Bragin et al., 2014; Kalsner and Chamberlain, 2015). We also found three patients carrying an extra copy of chromosome 21q (dup21q), which is associated with Down’s syndrome (DS#190685). Moreover, there were four other patients carrying 3.5 Mb deletion in the 17p11.2 (#40), 1.4 Mb deletion in the 7q11.23 (#107), 1.69 Mb deletion in the 17q12 (#59), and 1.9 Mb duplication in the 22q11.22q11.23 (#6) chromosomal regions (Table 1) that overlapped with the critical regions implicated with some well-characterized syndromes (Bragin et al., 2014), including Smith–Magenis syndrome (SMS# 182290), Williams–Beuren syndrome (WS# 194050), renal cysts and diabetes syndrome (RCAD#137920), and 22q11.2 distal deletion syndrome (OMIM#611867), respectively.

Interestingly, 2.83% (6/212) pathogenic CNVs in this cohort were found to be in the subtelomeric region (Table 1), which is within the range of the previously reported (2.4%–4.4%) diagnostic rate in patients with DD/ID and congenital anomaly (Ravnan et al., 2006; Shao et al., 2008). Of the six CNVs, we found 4.8 Mb deletion in the 4p16.3p16.2 (#1), 11.2 Mb duplication in the 17p13.3p12 (#79), and 8.6 Mb deletion in the 2q37.1q37.3 (#144) subtelomeric chromosomal regions that overlapped with the critical regions associated with the three well-characterized syndromes (Bragin et al., 2014) of Wolf–Hirschhorn syndrome (WHS#194190), Miller–Dieker syndrome (MDS#247200), and 2q37 monosomy (#600430), respectively. Subtelomeric regions are gene-rich and particularly prone to genomic instability due to the repeat sequences found in these areas. Mutation in these regions causes a variety of phenotypic abnormalities, particularly intellectual disability (ID) (Linardopoulou et al., 2005; Ruiter et al., 2007; Wu et al., 2010; Uddin et al., 2011; Rahman et al., 2019). Rahman et al. (2019) identified two siblings affected with ID who are both impacted by large terminal duplication (18.82 Mb) and deletion (3.90 Mb) CNVs. The remaining eight patients carried different lengths of deletion and duplication CNVs that disrupt the vital regions of the chromosome containing *bona fide* genes (*SCN1A*, *KANK1*, *DOCK8, etc.*) associated with different types of neurodevelopmental disorders (Nassir et al., 2021) (Table 1).

Identifying overlapping genes and pathways across disorders (Iourov et al., 2019; Zelenova et al., 2019) is critical to improving the understanding of their potential shared genetic etiology. Gene Ontology and KEGG pathway enrichment analysis of the impacted genes within the CNV breakpoints of all pathogenic deletions and duplication identified “ubiquitin-like protein transferase activity (GO:0019787),” “vesicle-mediated transport in synapse (GO:0099003),” “dependent protein catabolic process (GO:0030163),” “regulation of neuronal death (GO:1901214),” and “synaptic signaling (GO:0099536)” pathways to be highly significant (Figures 3A, B). Aberrations in autophagy-related (a major cellular catabolic process) gene mutations and signaling have been implicated in several neurodevelopmental disorders, including autism, tuberous sclerosis, Fragile X syndrome, and neurofibromatosis type 1 (Lee et al., 2013; Morice-Picard et al., 2016). Loss of function in the ubiquitin ligase gene *HERC2* has been associated with a severe neurodevelopmental phenotype (Morice-Picard et al., 2016). Further mutations in presynaptic genes have been linked to various neurodevelopmental disorders, including autism, ID (Zelenova et al., 2019), and epilepsy (Bonnycastle et al., 2020), and synaptic signaling has been identified as one of the principal molecular pathways affected in neurodevelopmental disorders (Parenti et al., 2020). Perturbations in the apoptotic signaling pathway have also been identified in various NDDs, including autism, Fragile X syndrome, and schizophrenia (Rudin and Thompson, 1997; Wei et al., 2014).

Analyses using the “critical-exon” method (Uddin et al., 2014, 2016) to identify constraint candidate genes discerned a high number of CEGs within the pathogenic variants (7.5) compared to VOUS (0.19) (*p* = 0.0002). Moreover, CEGs per pathogenic CNV were also previously reported (Wassman et al., 2019) as reflective of length bias and gene density of pathogenic variants. From the previous studies (Uddin et al., 2014; Uddin et al., 2016; Wassman et al., 2019), it is observed that critical exon genes are significantly enriched for *de novo* mutations in autism probands but not in unaffected siblings or in population control subjects. Identifying critical exon genes has shown (Wassman et al., 2019; Safizadeh Shabestari et al., 2022) improvement in clinical interpretations of rare CNVs. In this study, CEG analysis of short focal CNVs identified 18 unique CEGs highly expressed in the neurotypical human brain and which have a low burden of non-synonymous variants in the control population. To discover the role of these genes within the context of neurodevelopmental disorders and our cohort, we conducted a comprehensive literature search. For example, in our cohort, one autism patient (#20) was carrying a 476-kb pathogenic deletion disrupting the *PLCB1* gene. Girirajan et al. (2013) found an enrichment of microdeletions and duplications involving the *PLCB1* gene in individuals with autism. The other 17 genes were located within VOUS CNVs. Of the 17, we tried to find a common overlapping genomic breakpoint shared by multiple patients with similar clinical condition to identify candidate genes for the disrupted locus. In this cohort, we found two autism patients (#119 and #121) who harbored a common deletion breakpoint disrupting four genes (*SLC39A13*, *PSMC3*, *SPI1*, and *RAPSN)*, including one critical exon gene *PSMC3*. Autosomal recessive mutations in the *SLC39A13* gene are associated with a well-defined disease, Ehlers–Danlos syndrome, spondylodysplastic type 3 (OMIM# 612350). Mutation in the *SPI1* gene was previously reported in acute lymphoblastic leukemia (Seki et al., 2017). Autosomal recessive mutation in the *RAPSN* gene is associated with two other well-developed diseases, fetal akinesia deformation sequence 2 (#601592) and myasthenic syndrome, congenital, 11, associated with acetylcholine receptor deficiency (#616326). The other gene of the common breakpoint is *PSMC3* which encodes the 26S regulatory subunit 6A, also known as the 26S proteasome AAA-ATPase subunit (Rpt5) of the 19S proteasome complex responsible for recognition, unfolding, and translocation of substrates into the 20S proteolytic cavity of the proteasome (Tanaka, 2009). This suggests that *PSMC3* plays an essential role in the ubiquitin–proteasome system (UPS), which includes morphogenesis, dendritic spine structure, synaptic activity, and regulation of synaptic strength in neurons (Hegde, 2010; Tai et al., 2010; Bingol and Sheng, 2011; Hamilton and Zito, 2013). Recently, Kröll-Hermi et al. (2020) demonstrated that homozygous single-nucleotide variants in the *PSMC3* cause neurosensory syndrome combining deafness, cataract, and autism/neurodevelopmental delay due to proteotoxic stress. Furthermore, we found one 217-kb deletion (Landrum et al., 2016) (ClinVar_VCV000151005) and one 173-kb duplication (Bragin et al., 2014) (Decipher_412053) CNVs in two previously reported NDD patients in whom the *PSMC3* gene was also disrupted (Figures 4C, D; Supplementary Table S7). Although our patients did not show syndromic appearance at the age of 1.6 and 3.1 years, except ASD, we hypothesize on the basis of previous studies in UPS (Hegde, 2010; Tai et al., 2010; Bingol and Sheng, 2011; Hamilton and Zito, 2013), including cases in the DECIPHER (Bragin et al., 2014) database that heterozygous mutations in the *PSMC3* (size ∼7.7 kb) gene might be associated with NDDs without causing neurosensory syndrome.

In this study, we found a 20-year-old girl (#106), born in a consanguineous marriage with healthy parents, who was delivered preterm after an eventful pregnancy (IUGR). She had a history of delayed development. She had intellectual disability with dysmorphic features of an elongated face and long fingers (Supplementary Table S1). Her cognitive and physical conditions progressively worsened, starting at 15 years. At 18 years, she developed dyskinesia and speech and swallowing difficulties. Her MRI finding was normal, and she had no issues with walking or speech-related deficits. Analyzing the overlapping region of 14 previously reported deletions (length 0.19–4.91 Mb) (Dale et al., 2012; Gana et al., 2012; Bragin et al., 2014; Meyer et al., 2017; Zhao et al., 2018; Carecchio et al., 2019) and three duplications (length 3.31–12.63 Mb) (Bragin et al., 2014), our patient (#106) had a shorter 2.01 Mb duplication with a 38.4 kb (chr19:35,700,296-35,738,700) common overlapping region among the CNVs disrupting two genes, *ZBTB32* and *KMT2B* (Figure 5; Supplementary Table S8). Furthermore, CEG and GenomeArc analytics also identified *UBA2*, *USF2*, *SCN1B*, *KMT2B*, *COX6B1*, *LGI4*, and *ZNF599* genes.

*SCN1B* is known to be associated with atrial fibrillation, familial, 13 (#615377), Brugada syndrome 5 (#612838), developmental and epileptic encephalopathy 52 (#617350), and epilepsy with febrile seizures plus and type 1 (#604233) due to the presence of pathogenic mutations. Our patient had no history of seizures, epilepsy, or cardiac problems, indicating that *SCN1B* duplication might not be associated with the patient’s clinical condition. *KMT2B* is another interesting gene within this breakpoint where pathogenic mutations are associated with dystonia 28, childhood-onset (#617284) with core phenotypes described as limb-onset childhood dystonia that tends to spread progressively, resulting in generalized dystonia with craniocervical involvement. Co-occurring signs such as distinct facial dysmorphism and intellectual disability are most common (Meyer et al., 2017; Zech et al., 2019). A distinct group of *KMT2B* patients presented with neurodevelopmental disorders in the absence of dystonia or related movement disorders (Faundes et al., 2018; Zech et al., 2019; Cif et al., 2020). We also found some DECIPHER (Bragin et al., 2014) patients carrying a duplication CNV containing the *KMT2B* gene whose common phenotype was GDD in the absence of dystonia. Most of the clinical conditions of our patient (#106) in the form of global developmental delay, intrauterine growth retardation, intellectual disability, facial dysmorphism, dyskinesia, and speech and swallowing problems match with the *KMT2B*-related disorder. So, we hypothesize that *KMT2B* gene duplication in our patient might be associated with the *KMT2B*-related disorder. Although *KMT2B* haploinsufficiency due to frameshift (small insertion/deletion), non-sense, splice-site, missense, and large deletion mutations is the primary cause of disease mechanism (Meyer et al., 2017; Zech et al., 2019; Cif et al., 2020), it is also reported that the penetrance for *KMT2B-*related disease is high, with almost complete penetrance for protein-truncating variants and chromosomal deletions and reduced penetrance for missense variants (Zech et al., 2019; Cif et al., 2020). Within the common overlapping region, *ZBTB32* is another new candidate gene in our cohort with no previous report of association with dystonia or neurodevelopmental disorder patients.

In our cohort, a series of overlapping rare clinically relevant variants were identified in multiple patients (Supplementary Table S9). For example, a 96.8-Kb deletion was found in two unrelated ASD patients (#8 and #127) at 6p21.33, which disrupts the major histocompatibility complex (MHC) class I gene *MICA*, which was not previously implicated with broader neurodevelopmental disorder patients. Three other unrelated patients (#13, #15, and #207) carried 48 Kb duplication at 15q13.3, which disrupts the previously reported (Gillentine and Schaaf, 2015; Gillentine et al., 2017; Uddin M. et al., 2018) broader NDD genes *CHRNA7* and *OTUD7A*. We found a 381-Kb duplication in two unrelated ASD patients (#34 and #104) at 8q21.2, disrupting carbonic anhydrases II (*CA2*), a gene associated with the disease osteopetrosis, autosomal recessive 3, OPTB3 (OMIM# 259730). We found two siblings (#42 and #43) affected with variable NDD phenotypes and one unrelated patient (#152) carrying 233 kb–340 kb duplication at 3p26.3, disrupting the previously reported (Hu et al., 2015; Repnikova et al., 2020) broader NDD-associated gene *CNTN6*. We found two other unrelated ASD patients carrying 130 Kb duplication CNV, disrupting the previously reported (Yasuda et al., 2014) NDD gene *NPHP1*. Three unrelated patients (#57, #159, and #200) carried a 236-Kb overlapping focal duplicated region, disrupting the two constraint genes *NPY4R* and *GPRIN2*. In addition, we found two unrelated ASD patients (#65 and #99) carrying 51 Kb and 59 Kb duplication at 4p16.3, disrupting alpha-L-iduronidase, the *IDUA* gene.

## Conclusion

In this paper, we used multiple annotation tools (the “critical-exon” method and GenomeArc) to identify disease-associated candidate genes within rare CNVs. Although these tools can identify possible candidate genes, they may still miss some critical genes due to the limitations of the methods. We have shown the utility of chromosomal microarray analysis as a first-tier diagnostic technology for neurodevelopmental disorder patients. Without a proper genetic test, the clinical complexity alone may not be enough to identify the cause and often lead to a diagnostic odyssey. Although the price of microarray is reducing, it is still not affordable for most underdeveloped countries. This study shows how in near future developing countries can integrate such technology within their existing healthcare system. Despite the advantages of CMA, there are some notable limitations. Because only unbalanced CNVs are detected, arrays cannot identify balanced inversions/insertions or reciprocal translocations. Likewise, because of the overall resolution, arrays also will miss low-level mosaicism (typically below 20%) and point mutations or small insertions or deletions in single genes. Although there are limitations of CMA, it is true that this technology can precisely detect 10%–30% of NDD cases in a cost-effective way and will enable the detection of novel variants and genes from underrepresented populations.

## Data Availability

The original contributions presented in the study are included in the article/Supplementary Material; further inquiries can be directed to the corresponding author.
