# Supplementary material for: Construction of copy number variation landscape and characterization of associated genes in a Bangladeshi cohort of neurodevelopmental disorders
Source: Front Genet. 2023 Mar 7;14:955631. doi: 10.3389/fgene.2023.955631 (PMC10028086; doi:10.3389/fgene.2023.955631)
Supplement: Supplementary file 1 [file DataSheet1.PDF]

Supplementary Material

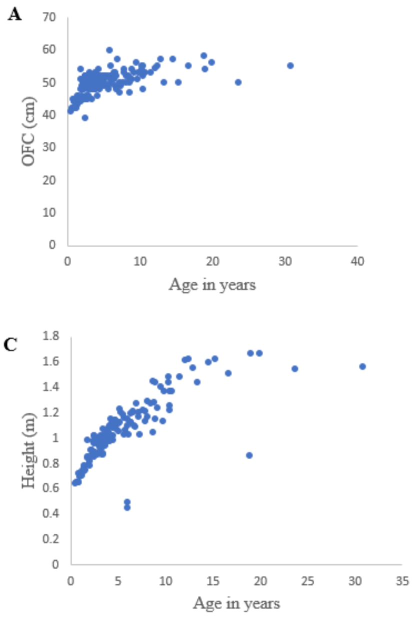

Supplementary Figure 1. OFC, Weight, Height and BMI distribution of 138 male patients. 96.38% (133/138) patients were children (<18 years) and only 3.62% (5/138) were adult (≥ 18 years).

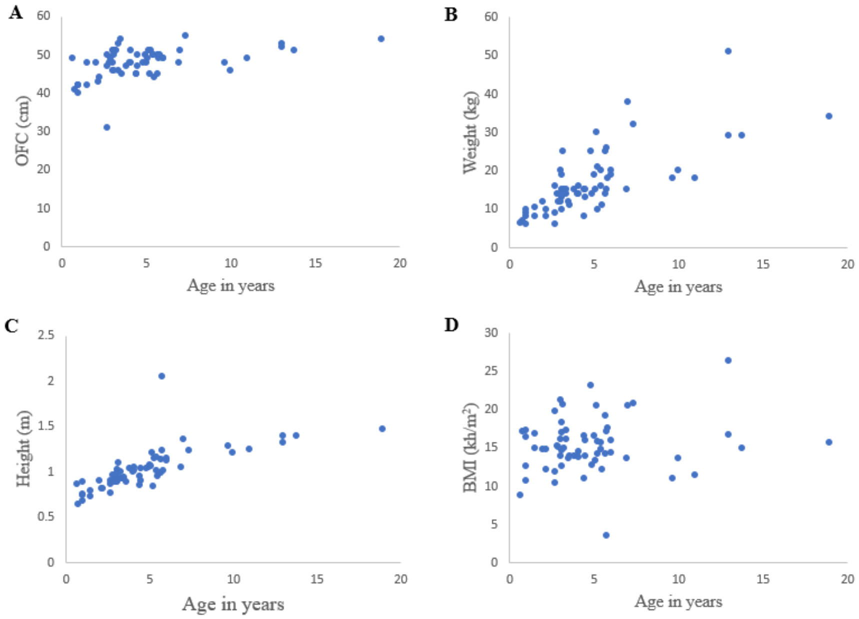

Supplementary Figure 2. OFC, Weight, Height and BMI distribution of 64 female patients. 98.44% (63/64) female patients were children (<18 years) and only 1.56% (1/64) was adult (≥ 18 years).

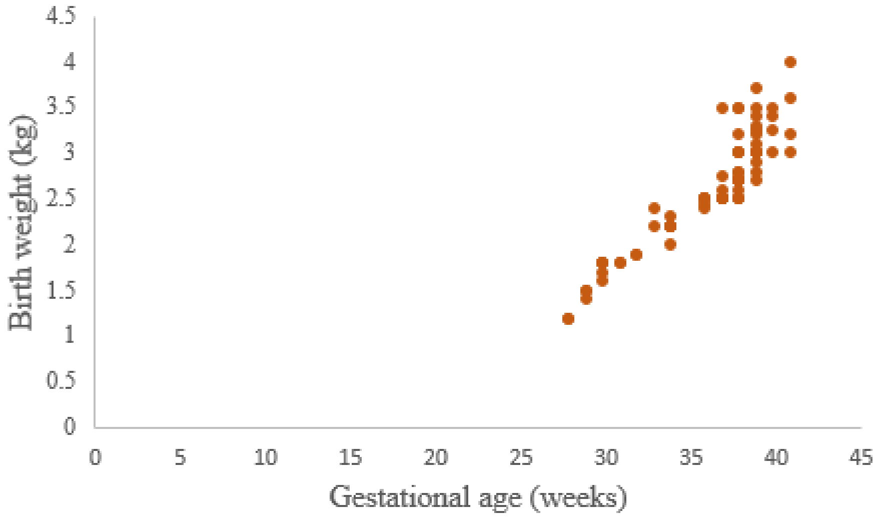

Supplementary Figure 3. Birth weight distribution of 76 patients. Of the 76, 68.42% (52/76) patients' birth weight was within normal range (2.5 to 4.5 kg)

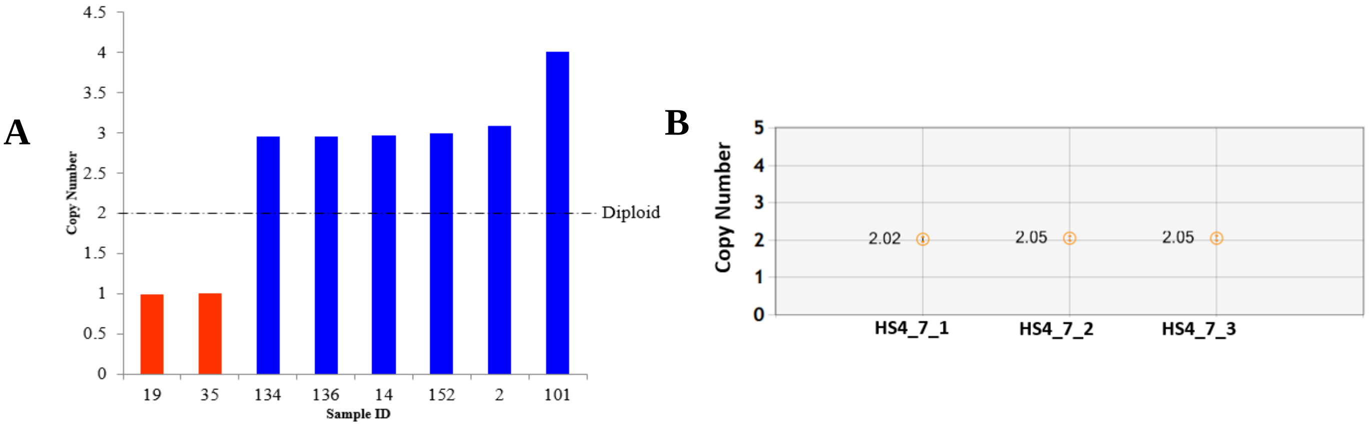

Supplementary Figure 4. ddPCR validation data. **(A)** Results showing confirm deletion and duplication CNVs in 8 pathogenic samples. Three probes (targeting SNHG14, TSPEAR and PKNOX2) were used and tested in three technical replicates. Y axis refers to number of copy identified in ddPCR and X axis refers to sample ID. Dot line indicates the normal copy of the corresponding variant in the healthy individuals. **(B)** ddPCR log intensity data shows there is no apparent deletion in that region.

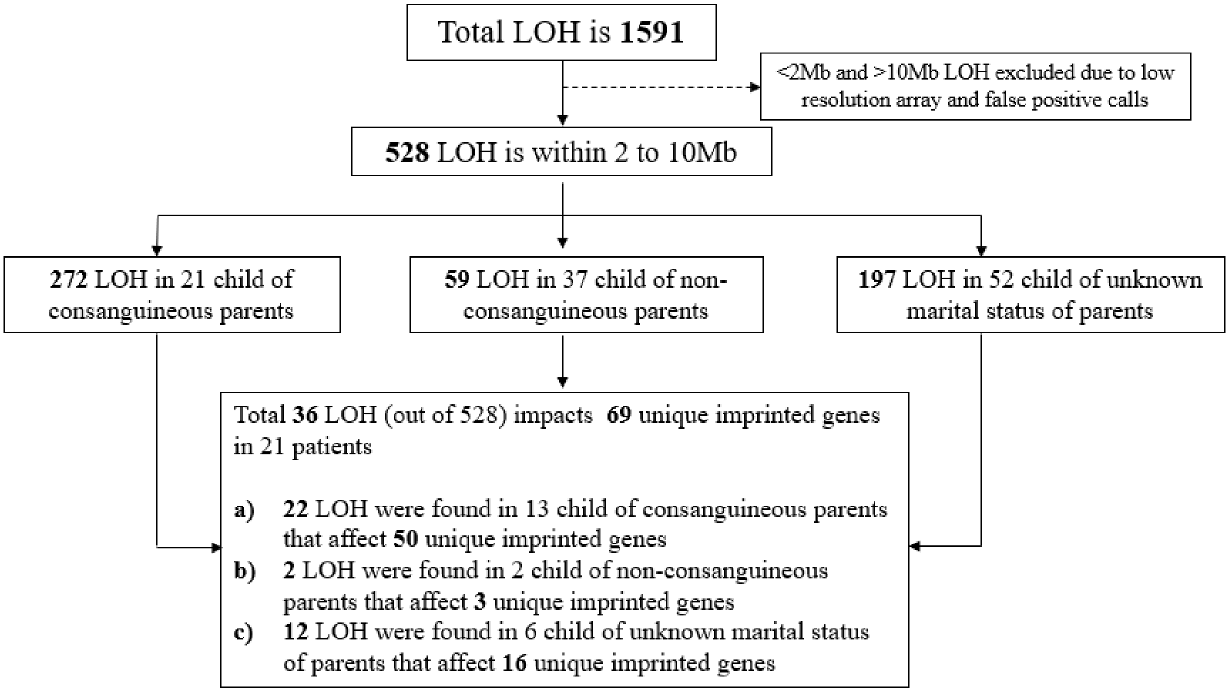

Supplementary Figure 5. Summary of Loss of Heterozygsity (LOH) identified in the cohort.

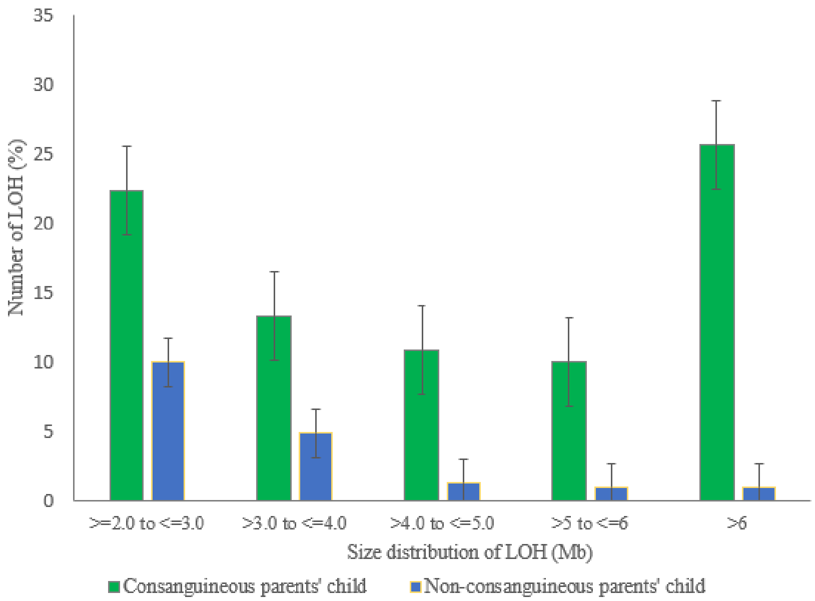

Supplementary Figure 6. Size distribution of 331 long LOH that are within 2 to 10Mb in the children of consanguineous and non-consanguineous parents. X-axis represents the size distribution of LOH and Y-axis represents the number of 331 LOH in percentage (%). Green bar indicates the size distribution of 272 LOH in the child of consanguineous parents and blue bar indicates the size distribution of 59 LOH present in the child of non-consanguineous parents. Due to lack of parental marital status of 52 child, size distribution of 197 LOH within 2 to 10MB was not included in this figure.

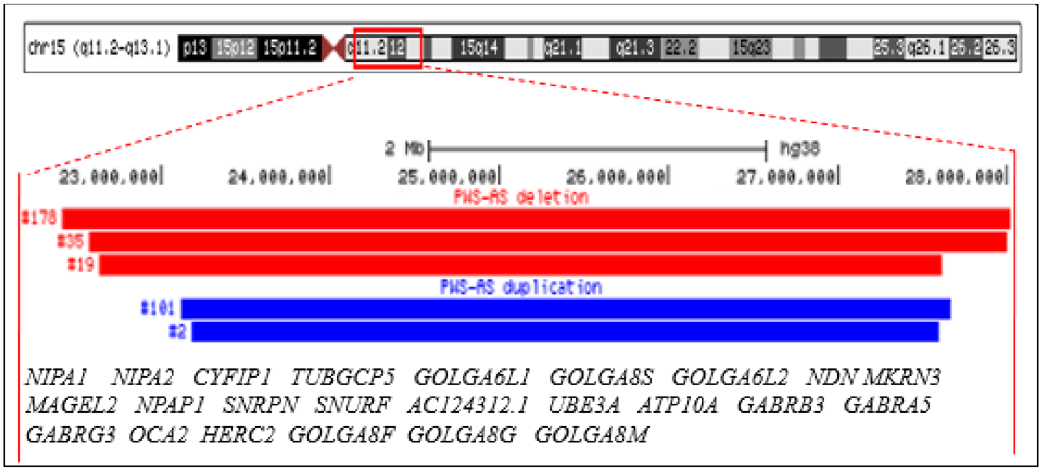

Supplementary Figure 7. A schematic representation of the overlapping CNVs in our patients (#2, #19, #35, #101 and #178). A close view of chromosome band 15q11.2q13.1 is displayed on the top. Red and blue rectangles symbolize gross deletion and duplication, respectively.
